# Stroke and cognitive impairment: understanding the connection and managing symptoms

**DOI:** 10.1097/MS9.0000000000001441

**Published:** 2023-11-01

**Authors:** Chukwuka Elendu, Dependable C. Amaechi, Tochi C. Elendu, Jennifer O. Ibhiedu, Emmanuel O. Egbunu, Afuh R. Ndam, Faith Ogala, Tunde Ologunde, Jesse C. Peterson, Adeyemo I. Boluwatife, Anietienteabasi O. Okongko, John O. Fatoye, Otite L. Akpovona, Samuel O. Onyekweli, Afolabi Y. Temitope, Abraham O. Achimugu, Adekola V. Temilade

**Affiliations:** a Federal Medical Center; b Imo State University, Owerri, Imo State; c Igbinedion University Okada, Edo State; d Babcock University; e Sacred Heart Hospital, Abeokuta; f Rufina Catholic Medical Center, Iperu, Ogun State; g University of Nigeria, Enugu State; h Lily Hospitals; i Westend Hospital, Warri, Delta State; j University of Ilorin, Ilorin, Kwara State; k Obafemi Awolowo University, Osun State; l Lautech Teaching Hospital, Ogbomoso, Oyo State; m Federal Medical Centre, Bida, Federal Capital Territory, Nigeria; n The Manor Hospital, Oxford; o King’s College Hospital NHS Foundation Trust, Denmark Hill, London, UK

**Keywords:** cognitive impairment, connection, neuroimaging, stroke, TOAST classification

## Abstract

Stroke is a leading cause of long-term disability worldwide, and cognitive impairment is a common consequence of stroke. Understanding the connection between stroke and cognitive impairment is crucial for effectively managing symptoms and improving patients’ quality of life. This abstract provides an overview of the relationship between stroke and cognitive impairment and explores strategies for managing cognitive symptoms in stroke survivors. A comprehensive review of relevant literature was conducted to examine the association between stroke and cognitive impairment. Various factors contributing to cognitive impairment after stroke were explored, including the location and severity of the stroke, vascular risk factors, and underlying mechanisms. Evidence-based strategies for managing cognitive symptoms in stroke survivors were also analyzed, such as cognitive rehabilitation, pharmacological interventions, and lifestyle modifications. The review revealed a strong link between stroke and cognitive impairment. The location and severity of the stroke play a significant role in determining the specific cognitive deficits experienced by individuals. Vascular risk factors, including hypertension, diabetes, and atrial fibrillation, contribute to cognitive decline after stroke. Mechanisms such as cerebral hypoperfusion, white matter damage, and neuroinflammation also play a role. Cognitive rehabilitation programs have shown promising results in improving cognitive function, while certain medications may help manage specific cognitive symptoms. Lifestyle modifications like physical exercise and a healthy diet have been associated with better cognitive outcomes in stroke survivors.

## Introduction and background

Highlights

- Understanding the connection: This article explores the intricate relationship between stroke and cognitive impairment, shedding light on how strokes can lead to cognitive decline and impact brain function.
- Impact on cognitive function: The article highlights the specific areas of cognitive function affected by strokes, such as memory, attention, language, and executive functions, helping readers grasp the scope of cognitive impairment resulting from stroke.
- Risk factors and prevention: It discusses the key risk factors for stroke-related cognitive impairment, emphasizing the importance of stroke prevention strategies to reduce the likelihood of cognitive decline.
- Rehabilitation and management: The article offers insights into effective rehabilitation and management techniques to address cognitive symptoms post-stroke, empowering readers with practical strategies for coping and recovery.
- Empowering patients and caregivers: It delivers a crucial clinical message, stressing the significance of early detection, prompt treatment, and support for stroke survivors and their caregivers to optimize cognitive outcomes and overall quality of life.

Stroke is a significant health concern worldwide, representing a leading cause of long-term disability and mortality^1^. Cognitive impairment refers to a broad range of deficits in cognitive function, including memory, attention, executive function, language, and visuospatial skills^2,3^. Understanding the connection between stroke and cognitive impairment is crucial for effectively managing symptoms and improving stroke survivors’ quality of life.

Numerous studies have demonstrated a strong association between stroke and cognitive impairment^4^. The cognitive deficits experienced by stroke survivors can profoundly impact their daily functioning, independence, and overall well-being^5^.

Several factors contribute to cognitive impairment after stroke. The location and severity of the stroke play a critical role in determining the specific cognitive deficits observed in individuals^6^. For instance, strokes affecting the frontal lobes are often associated with executive dysfunction, while strokes involving the temporal lobes can lead to memory impairment^7^. Vascular risk factors, such as hypertension, diabetes, and atrial fibrillation (AF), are also associated with an increased risk of cognitive decline after stroke^8^. Moreover, underlying mechanisms, including cerebral hypoperfusion, white matter damage, and neuroinflammation, contribute to cognitive impairment in stroke survivors^9^.

Effective management of cognitive symptoms in stroke survivors is essential for maximizing functional recovery and improving quality of life. Cognitive rehabilitation programs have shown promise in addressing specific deficits and enhancing cognitive function^10^. These programs typically involve targeted exercises and strategies to improve attention, memory, problem-solving, and other cognitive abilities. Pharmacological interventions may also play a role in managing cognitive symptoms. Medications such as cholinesterase inhibitors have been investigated for their potential to improve cognition in stroke survivors^11^. In addition to these interventions, lifestyle modifications, including regular physical exercise and a healthy diet, have been associated with better cognitive outcomes in stroke survivors^12^.

This paper overviews the relationship between stroke and cognitive impairment, exploring the underlying mechanisms and risk factors. It also discusses evidence-based strategies for managing cognitive symptoms in stroke survivors. By understanding the connection between stroke and cognitive impairment and implementing appropriate interventions, healthcare professionals can enhance the rehabilitation process and improve the long-term outcomes for stroke survivors.

### Objectives of the study

#### To examine the association between stroke and cognitive impairment

The primary objective is to investigate the relationship between stroke and cognitive impairment. We analyze relevant literature and research findings to determine the prevalence and extent of cognitive deficits in stroke survivors.

#### To identify factors contributing to cognitive impairment after stroke

Another objective is to identify the factors contributing to cognitive impairment following a stroke. We will explore variables such as the location and severity of the stroke, vascular risk factors, and underlying mechanisms, including cerebral hypoperfusion, white matter damage, and neuroinflammation.

#### To explore strategies for managing cognitive symptoms in stroke survivors

This study aims to investigate evidence-based strategies for managing cognitive symptoms in stroke survivors. We will analyze interventions such as cognitive rehabilitation programs, pharmacological treatments, and lifestyle modifications to determine their effectiveness in improving this population’s cognitive function and quality of life.

#### To understand the impact of cognitive impairment on stroke outcomes

The study also aims to examine the effects of cognitive impairment on stroke outcomes, including functional recovery, post-stroke complications, and long-term prognosis. By understanding the implications of cognitive impairment, we can better tailor interventions to optimize stroke recovery and patient outcomes.

#### To provide recommendations for clinical practice and future research

Based on the findings of this study, our objective is to offer practical guidance for healthcare professionals in managing cognitive impairment in stroke survivors. Additionally, we aim to identify gaps in knowledge and highlight areas for future research, including developing personalized treatment approaches and exploring novel interventions for cognitive rehabilitation.

By addressing these objectives, this study aims to contribute to a deeper understanding of the connection between stroke and cognitive impairment and provide insights into practical strategies for managing cognitive symptoms in stroke survivors.

## Methodology

### Literature search and selection

#### Database search

A comprehensive search of electronic databases, including PubMed, MEDLINE, and Google Scholar, was conducted. The search terms included “stroke”, “cognitive impairment”, “cognitive deficits”, “cognitive dysfunction”, “cognitive symptoms”, and related variations. Boolean operators (AND, OR) effectively combined the search terms.

#### Inclusion and exclusion criteria

Studies were included if they met the following criteria: (a) focused on stroke survivors, (b) examined the relationship between stroke and cognitive impairment, (c) provided information on the factors contributing to cognitive impairment, and (d) discussed strategies for managing cognitive symptoms. Studies were excluded if they were not published in English or lacked sufficient relevance to the topic.

#### Study selection

Two independent reviewers screened the titles and abstracts of the identified articles to determine their eligibility for full-text review. Discrepancies were resolved through discussion and consensus. The full texts of potentially relevant articles were then assessed for final inclusion in the study.

### Data extraction and synthesis

#### Data extraction

A standardized data extraction form was developed to capture relevant information from the selected studies. Data extraction included study characteristics (e.g. authors, publication year), study design, participant characteristics, stroke subtype, cognitive assessment measures, and key findings related to the study’s objectives.

#### Data synthesis

The extracted data were synthesized to provide a comprehensive overview of the association between stroke and cognitive impairment, factors contributing to cognitive deficits, and strategies for managing cognitive symptoms. Key themes and patterns were identified and summarized to address the research objectives.

### Quality assessment

#### Quality criteria

The included studies’ methodological quality and risk of bias were assessed using appropriate quality assessment tools, such as the Newcastle–Ottawa Scale for observational studies and the Cochrane Risk of Bias Tool for randomized controlled trials. The assessment criteria included study design, sample size, participant selection, outcome assessment, and statistical analysis.

#### Quality assessment process

Two independent reviewers assessed the quality of the included studies. Any discrepancies in the quality assessment were resolved through discussion and consensus.

### Analysis and reporting

#### Narrative synthesis

A narrative synthesis approach was used to summarize the findings of the included studies. The results were organized according to the research objectives, addressing the association between stroke and cognitive impairment, factors contributing to cognitive deficits, and strategies for managing cognitive symptoms.

#### Subgroup analysis

Where applicable, subgroup analysis was conducted based on stroke subtype, severity, or other relevant characteristics to explore potential variations in the relationship between stroke and cognitive impairment.

#### Ethical considerations

Ethical approval was not required as this study is based on a comprehensive review of published literature. The study adhered to established guidelines for systematic reviews and meta-analyses, ensuring transparency and rigor in the methodology.

This methodology aims to comprehensively analyze the association between stroke and cognitive impairment, identify contributing factors, and propose strategies for managing cognitive symptoms in stroke survivors.

### Definition and epidemiology

Stroke is a significant global health burden in terms of morbidity and mortality. Approximately 15 million people are estimated to experience a stroke each year worldwide^1^. The incidence and prevalence of stroke vary across different populations and regions. Age is a critical risk factor for stroke, with the incidence increasing significantly with advancing age^2^.

The Global Burden of Disease Study reported that stroke was the second leading cause of death globally in 2013, accounting for 11.8% of all deaths^1^. However, there has been a decline in stroke-related mortality in recent years, particularly in high-income countries, attributed to advancements in acute stroke management and improved access to specialized stroke care^2^.

Cognitive impairment is a common consequence of stroke, affecting many stroke survivors. The prevalence of post-stroke cognitive impairment varies depending on the population studied and the assessment methods used. Studies have reported that cognitive impairment can occur in ~30–70% of stroke survivors^4^. The variability in prevalence can be attributed to factors such as the severity and location of the stroke, comorbidities, and the assessment tools employed.

Various stroke characteristics contribute to the risk of cognitive impairment. The location of the stroke plays a crucial role in determining the specific cognitive deficits experienced by individuals. For example, strokes involving the frontal lobes are often associated with executive dysfunction, while strokes affecting the temporal lobes can lead to memory impairment^8^. The severity of the stroke is also a significant factor, with larger and more severe strokes generally associated with a higher risk of cognitive impairment.

Vascular risk factors significantly contribute to cognitive decline after stroke. Hypertension, diabetes mellitus, AF, and hyperlipidemia are well-established risk factors for stroke and cognitive impairment^13^. These risk factors contribute to the underlying vascular pathology that can damage brain structures, impair cerebral blood flow, and lead to cognitive deficits.

Age, educational level, and pre-existing cognitive impairments are also associated with the risk of cognitive decline after stroke. Older age and lower educational attainment have been consistently linked to a higher risk of post-stroke cognitive impairment^9^. Pre-existing cognitive impairments or conditions such as mild cognitive impairment or dementia are also significant predictors of cognitive decline after stroke.

Understanding the epidemiology of stroke and cognitive impairment is crucial for effective healthcare planning, resource allocation, and developing preventive and management strategies. By identifying the risk factors and prevalence rates, healthcare professionals can better target interventions and support stroke survivors appropriately.

### Pathophysiology

The pathophysiology of cognitive impairment following stroke is complex and involves various mechanisms contributing to brain structure and function disruption. Understanding the underlying pathophysiological processes is crucial for developing effective strategies to manage cognitive symptoms in stroke survivors.

#### Cerebral infarction and ischemic cascade

Ischemic stroke, which accounts for the majority of stroke cases, occurs when there is a blockage of blood flow to a specific brain region, leading to tissue damage. Cerebral infarction results in the deprivation of oxygen and glucose, triggering a cascade of pathological events. The ischemic cascade involves excitotoxicity, oxidative stress, inflammation, and apoptosis, leading to neuronal cell death and tissue damage^14^.

#### Vascular factors

Vascular risk factors, such as hypertension, diabetes mellitus, and atherosclerosis, contribute to the pathophysiology of cognitive impairment in stroke. These risk factors lead to endothelial dysfunction, vascular remodeling, and reduced cerebral blood flow, impairing the delivery of nutrients and oxygen to brain regions and promoting cerebral hypoperfusion. Chronic cerebral hypoperfusion contributes to white matter lesions, microinfarcts, and small vessel disease associated with cognitive impairment^15^.

#### Neuroinflammation

In response to cerebral ischemia, inflammatory processes are activated within the brain. Activated microglia and astrocytes release pro-inflammatory cytokines, such as interleukin-1β (IL-1β), tumor necrosis factor-alpha (TNF-α), and interleukin-6 (IL-6). Neuroinflammation exacerbates neuronal damage, disrupts synaptic connections, and contributes to neurodegeneration, ultimately leading to cognitive impairment^16^.

#### White matter damage

Ischemic stroke can damage white matter, which is significant in cognitive impairment. Axonal injury, demyelination, and disruption of white matter tracts impair neuronal connectivity and communication. White matter lesions, such as leukoaraiosis and lacunar infarcts, are frequently observed in post-stroke cognitive impairment and contribute to executive dysfunction, information processing deficits, and slowed cognitive speed^17^.

#### Neurodegeneration and protein accumulation

Stroke can trigger neurodegenerative processes, including the accumulation of abnormal protein aggregates, such as amyloid-beta (Aβ) plaques and tau tangles, similar to those observed in Alzheimer’s disease (AD). These pathological hallmarks in stroke survivors can contribute to cognitive impairment and increase the risk of developing mixed dementia, characterized by vascular and neurodegenerative pathology^18^.

#### Disruption of neurotransmitter systems

Stroke can disrupt various neurotransmitter systems, leading to imbalances that contribute to cognitive impairment. Reductions in acetylcholine, a key neurotransmitter involved in learning and memory, are commonly observed after stroke and contribute to cognitive deficits. Alterations in other neurotransmitters, including dopamine, serotonin, and norepinephrine, can also impact cognitive function^19^.

#### Secondary complications

Stroke can lead to secondary complications, such as seizures, infections, and mood disorders, further contributing to cognitive impairment. Seizures, particularly in the acute phase, can cause neuronal damage and worsen cognitive outcomes. Post-stroke depression and anxiety can negatively affect cognitive function, attention, and motivation, further exacerbating cognitive impairment^20^.

Understanding the multifaceted pathophysiology of cognitive impairment after stroke provides insights into potential targets for interventions and therapeutic strategies. Targeting specific mechanisms involved in neuroinflammation, oxidative stress, white matter integrity, and neurotransmitter imbalances may mitigate cognitive decline and improve outcomes for stroke survivors.

### Investigations and diagnosis

Diagnosing cognitive impairment following a stroke involves a comprehensive evaluation that includes clinical assessments, cognitive testing, neuroimaging, and laboratory investigations. These investigations aim to identify the presence and severity of cognitive deficits, determine potential underlying causes, and guide treatment planning for stroke survivors.

#### Clinical assessment

A detailed medical history and physical examination are essential to the diagnostic process. The medical history should focus on identifying risk factors for stroke and cognitive impairment, including hypertension, diabetes, previous strokes, and neurodegenerative disorders. The physical examination may help identify signs of focal neurological deficits or other conditions that could contribute to cognitive impairment.

#### Cognitive testing

Various cognitive assessment tools are available to evaluate different cognitive domains and detect cognitive impairment. The Mini-Mental State Examination (MMSE) and Montreal Cognitive Assessment (MoCA) are screening tests that assess multiple cognitive functions, including attention, memory, language, and executive function. More extensive neuropsychological testing may be necessary to provide a detailed profile of cognitive strengths and weaknesses^21^.

#### Neuroimaging

Neuroimaging plays a crucial role in the diagnosis of stroke and the evaluation of cognitive impairment. Structural brain imaging techniques, such as magnetic resonance imaging (MRI) and computed tomography (CT) scans, can detect acute and chronic brain lesions, assess the location and extent of the stroke, and identify other structural abnormalities, such as white matter lesions. Functional neuroimaging techniques, including positron emission tomography (PET) and functional MRI (fMRI), can provide additional insights into brain activity and connectivity in individuals with cognitive impairment^22^.

#### Laboratory investigations

Laboratory investigations are essential to identify potential underlying causes of cognitive impairment, such as metabolic abnormalities, inflammatory markers, or genetic factors. Blood tests may include complete blood count (CBC), liver and kidney function tests, lipid profiles, glucose levels, and thyroid function tests. Genetic testing may be considered in specific cases, particularly when a hereditary cause of cognitive impairment is suspected^23^.

#### Neuropsychiatric evaluation

A comprehensive assessment of neuropsychiatric symptoms, including depression, anxiety, and psychosis, is essential in individuals with cognitive impairment. Standardized scales and questionnaires, such as the Geriatric Depression Scale (GDS) or Hospital Anxiety and Depression Scale (HADS), can help identify and quantify these symptoms. A psychiatric evaluation may be warranted to assess the impact of mood disorders on cognitive function and guide appropriate treatment interventions^24^.

The combination of these investigations helps establish the diagnosis of cognitive impairment following a stroke, determine the severity and specific cognitive deficits, and identify potential underlying causes. The findings from these assessments guide treatment planning and the development of personalized interventions to manage cognitive symptoms, promote functional recovery, and enhance the quality of life for stroke survivors.

## Management

Managing cognitive impairment following stroke involves a multidisciplinary approach to address the underlying causes and the cognitive symptoms. The management strategies focus on cognitive rehabilitation, pharmacological interventions, addressing modifiable risk factors, and supporting stroke survivors and their caregivers.

### Cognitive rehabilitation

Cognitive rehabilitation programs are essential for managing cognitive impairment after stroke. These programs employ various techniques and strategies to improve cognitive function, promote compensatory strategies, and enhance functional outcomes. Cognitive rehabilitation can include individualized interventions targeting specific cognitive domains, such as attention, memory, language, and executive functions. The programs may involve computer-based training, behavioral therapies, environmental modifications, and psychoeducation for stroke survivors and their families^25^.

Cognitive rehabilitation involves therapeutic techniques and exercises tailored to the individual’s specific cognitive deficits. It is typically led by a team of healthcare professionals, including neuropsychologists, occupational therapists, speech–language pathologists, and physical therapists. The program is personalized to target the cognitive challenges the stroke survivor faces.

The process of cognitive rehabilitation typically includes the following steps:

1. Assessment: The healthcare team thoroughly evaluates the specific cognitive deficits and their impact on daily activities. This evaluation helps in creating a tailored rehabilitation plan.
2. Goal setting: Based on the assessment, specific goals are set for the individual. These goals aim to improve cognitive functions, enhance daily living skills, and promote independence.
3. Cognitive training: This aspect involves various exercises and activities that target impaired cognitive functions. For example, memory training may include strategies for improving recall or mnemonic techniques.
4. Compensatory strategies: Alongside cognitive training, individuals are taught compensatory strategies to cope with cognitive challenges. These strategies include using memory aids, visual cues, or setting reminders.
5. Environmental modifications: Adjusting the physical environment can facilitate better cognitive functioning. This may involve organizing living spaces, reducing distractions, or implementing assistive technology.
6. Repetition and practice: Consistent practice and repetition of cognitive exercises are essential for brain plasticity and long-term improvement.
7. Monitoring and feedback: Throughout the rehabilitation process, the healthcare team closely monitors progress and provides feedback to make necessary adjustments to the program.
8. Integration into daily life: The ultimate goal of cognitive rehabilitation is to enable individuals to apply the learned skills and strategies in their daily activities, promoting greater independence and a higher quality of life.

It is important to note that cognitive rehabilitation may not entirely restore all pre-stroke cognitive abilities. Still, it can lead to significant improvements and help individuals adapt to their cognitive changes effectively.

### Pharmacological interventions

No specific pharmacological treatment for post-stroke cognitive impairment is currently approved. However, certain medications have been investigated for their potential benefits. Cholinesterase inhibitors, such as donepezil, have been studied and may show modest improvements in cognitive function in some individuals with post-stroke cognitive impairment^26^. Other medications, such as memantine, have been explored, but the evidence for their effectiveness remains limited. The use of pharmacological interventions should be individualized based on the specific needs and risks of each patient.

### Management of modifiable risk factors

Addressing modifiable risk factors is crucial for preventing further cognitive decline and promoting brain health in stroke survivors. Management of vascular risk factors, including hypertension, diabetes, dyslipidemia, and smoking cessation, is essential to reduce the risk of recurrent strokes and slow down the progression of cognitive impairment. Lifestyle modifications, such as regular physical activity, a healthy diet, weight management, and stress reduction, also significantly manage cognitive symptoms^27^.

### Supportive care and education

Providing comprehensive support and education to stroke survivors and their caregivers is crucial. Education about stroke, cognitive impairment, and available resources can help individuals and their families understand the condition, manage expectations, and engage in self-care. Support groups, counseling, and community services can provide emotional support, practical guidance, and an opportunity to share experiences with others facing similar challenges^26^.

### Monitoring and follow-up

Regular monitoring and follow-up are essential to assess the effectiveness of interventions, track cognitive changes, and adjust the management plan accordingly. Periodic cognitive assessments and functional evaluations can help identify any deterioration or improvement in cognitive function and guide further interventions or modifications in the management plan^27^.

Managing cognitive impairment following stroke requires a holistic and individualized approach, considering each patient’s needs and circumstances. Collaborative efforts among healthcare professionals, including neurologists, neuropsychologists, rehabilitation specialists, and primary care providers, are crucial to ensure comprehensive care and optimizing outcomes for stroke survivors.

### TOAST classification of stroke

The TOAST (Trial of Org 10172 in Acute Stroke Treatment) classification is widely used for categorizing ischemic strokes based on their etiology. It provides valuable information for determining the underlying cause of the stroke, guiding treatment decisions, and predicting prognosis. The TOAST classification system divides ischemic strokes into five significant subtypes:

#### Large-artery atherosclerosis (LAA)

This subtype is characterized by atherosclerotic plaques in the large arteries that supply the brain. The plaques can lead to stenosis or occlusion, reducing blood flow to specific brain regions. Diagnostic criteria for LAA include imaging evidence of significant arterial stenosis or occlusion in combination with clinical features consistent with the involved artery territory^28^.

#### Cardioembolism (CE)

Cardioembolic strokes occur when a blood clot or other debris originating from the heart or proximal arteries travels to the brain, causing occlusion of cerebral arteries. Familiar sources of cardiac emboli include AF, myocardial infarction, valvular heart disease, and intracardiac thrombi. The diagnosis of CE requires identifying a potential cardiac source of emboli and the clinical features of stroke^28^.

#### Small-vessel occlusion (SVO)

SVO, also known as lacunar infarction, involves the occlusion of small penetrating arteries that supply deep brain structures. The most common underlying cause of SVO is lipohyalinosis, characterized by thickening and hyalinization of the small vessel walls. Lacunar infarctions are often associated with vascular risk factors, such as hypertension and diabetes mellitus. Imaging findings consistent with lacunar infarcts, such as small, deep infarcts on MRI, are crucial for diagnosing SVO^28^.

#### Stroke of other determined etiology (SOE)

This category includes ischemic strokes with a well-defined cause other than the three previously mentioned subtypes. Examples include stroke associated with non-atherosclerotic vasculopathy, such as arterial dissection or vasculitis, and stroke related to hematological disorders, such as sickle cell disease or coagulopathies. The diagnosis of SOE requires specific diagnostic criteria based on the underlying etiology^28^.

#### Stroke of undetermined etiology (SUE)

SUE refers to ischemic strokes for which the underlying cause remains uncertain despite thorough evaluation. These cases may lack identifiable vascular lesions or specific cardioembolic sources; other potential etiologies have been excluded. The diagnosis of SUE is made when the assessment does not reveal sufficient evidence to assign the stroke to one of the different TOAST subtypes^28^.

The TOAST classification system provides a standardized approach to classifying ischemic strokes based on their etiology. It assists clinicians in determining appropriate treatment strategies, such as anticoagulation for cardioembolic strokes or risk factor management for atherosclerotic strokes. Additionally, it helps predict prognosis and guide secondary prevention strategies to reduce the risk of recurrent strokes.

### Prognosis

The prognosis of stroke, including cognitive impairment and functional outcomes, can vary widely depending on various factors, including the type of stroke, the severity of the initial event, comorbidities, age, and individual characteristics. Understanding the prognosis following a stroke is essential for appropriate treatment planning, patient counseling, and rehabilitation interventions.

#### Mortality

Stroke is a leading cause of mortality worldwide. The mortality rate following a stroke depends on several factors, including the type and location of the stroke, the presence of comorbidities, and the severity of the initial event. Hemorrhagic strokes, particularly intracerebral hemorrhages, are associated with higher mortality rates than ischemic strokes^29^. Older age, male gender, and the presence of multiple comorbidities further increase the risk of mortality following a stroke.

#### Functional outcome

A stroke can result in various functional impairments, including motor deficits, sensory loss, language difficulties, and cognitive impairment. The extent of brain damage influences the practical outcome following a stroke, the brain area affected, and the effectiveness of rehabilitation interventions. The degree of functional recovery can vary widely among individuals, with some experiencing significant improvements while others may have persistent disabilities^30^.

#### Recurrence of stroke

Stroke survivors are at an increased risk of recurrent strokes. The risk of recurrence depends on several factors, including the initial stroke’s underlying cause, modifiable risk factors, and the implementation of secondary prevention measures. Effective management of vascular risk factors, such as hypertension, diabetes, and dyslipidemia, along with appropriate use of anticoagulation in high-risk individuals, can significantly reduce the risk of recurrent strokes^31^.

#### Cognitive impairment

Cognitive impairment is a common consequence of stroke and can significantly impact long-term prognosis and quality of life. The presence of cognitive impairment following a stroke is associated with increased disability, dependence on activities of daily living, and higher rates of institutionalization. The severity and specific cognitive deficits can vary among individuals, with some experiencing mild impairments while others developing more severe dementia-like symptoms^32^.

#### Quality of life

Stroke can profoundly impact an individual’s quality of life. The physical, cognitive, and emotional consequences of stroke can result in reduced functional independence, social isolation, and decreased overall well-being. Rehabilitation interventions, including physical therapy, occupational therapy, and cognitive rehabilitation, play a crucial role in improving functional outcomes and enhancing the quality of life for stroke survivors^33^.

It is important to note that individual prognosis following a stroke is highly variable, and generalizations should be made cautiously. Numerous factors can influence the prognosis, and it is essential to consider each stroke survivor’s specific characteristics and needs. A multidisciplinary approach, including close collaboration between healthcare providers, rehabilitation specialists, and caregivers, is necessary to optimize functional outcomes, support recovery, and improve overall prognosis.

### Comorbidities

Comorbidities are pre-existing medical conditions that often coexist with stroke and can influence its occurrence, severity, treatment outcomes, and prognosis. Managing comorbidities is crucial in stroke management to optimize patient care and improve overall results. Here are some common comorbidities associated with stroke:

#### Hypertension

Increased blood pressure is a significant risk factor for stroke and cognitive impairment. Uncontrolled hypertension can damage blood vessels, increasing the likelihood of strokes. Additionally, it can cause cerebral microbleeds and small vessel disease, which can contribute to cognitive decline. Managing hypertension through lifestyle changes and medications may help reduce stroke and cognitive impairment risk^34^.

#### Diabetes mellitus

Diabetes is associated with a high risk of stroke and vascular-related cognitive impairment. Increased blood sugar levels can damage blood vessels, reducing blood flow to the brain. This can result in cognitive deficits, including memory problems and decreased cognitive processing speed. Managing diabetes through proper glucose control, diet, exercise, and medication can help mitigate the risk of cognitive impairment^35^.

#### Atrial fibrillation

AF is an irregular heartbeat that can lead to blood clots, which can cause ischemic strokes. Strokes resulting from AF can lead to cognitive impairment, primarily if they affect critical brain regions. Managing AF with anticoagulant medications and other treatments can help prevent strokes and associated cognitive decline^36^.

#### Dyslipidemia

High cholesterol levels can lead to atherosclerosis, narrowing blood vessels and increasing the likelihood of strokes that impact cognition. Lifestyle changes and medication can help manage dyslipidemia and reduce cognitive risks^37^.

#### Obesity

Excess weight can increase the risk of hypertension, diabetes, and cardiovascular disease, all contributing to cognitive impairment. Weight management and a healthy lifestyle are essential for reducing cognitive risks^38^.

#### Smoking

Smoking is a modifiable risk factor for stroke and cognitive decline. It can damage blood vessels, increase inflammation, and reduce blood flow to the brain, leading to brain cell damage and cognitive impairment. Quitting smoking can significantly improve vascular health and reduce the risk of stroke and cognitive decline^39^.

#### Cardiovascular disease

Conditions like heart disease and a history of heart attacks increase the likelihood of strokes and vascular damage in the brain, leading to cognitive problems. Preventive measures like lifestyle changes and medication can help manage cognitive risks^8^.

#### Chronic kidney disease

Impaired kidney function can lead to hypertension and other vascular issues, contributing to cognitive decline. Managing kidney disease and its associated risk factors can be crucial for cognitive health^40^.

Effective management of cognitive impairment involves identifying and addressing these risk factors early on. Lifestyle modifications such as maintaining a balanced diet, regular exercise, and managing stress can improve overall vascular health and cognitive function. Additionally, medical interventions and medications targeted at controlling the underlying risk factors can help reduce the impact of stroke on cognitive abilities.

It is essential for individuals at risk or already experiencing cognitive impairment to work closely with healthcare professionals to develop personalized treatment plans that address the specific risk factors contributing to their condition. Early intervention and comprehensive management can significantly improve outcomes and quality of life.

### Roles of risk factors in cognitive decline

The roles of risk factors are crucial in understanding the connection between stroke and cognitive impairment. Some key risk factors include:

#### Age

Advancing age is a significant risk factor for cognitive decline and vascular-related disorders.

#### Hypertension (high blood pressure)

Uncontrolled high blood pressure can damage brain blood vessels, increasing the risk of cognitive impairment.

#### Diabetes

Poorly managed diabetes can harm blood vessels and nerves in the brain, contributing to cognitive decline.

#### Atrial fibrillation

AF, a heart rhythm disorder, can lead to blood clots, increasing the likelihood of strokes that can cause cognitive impairment.

#### Smoking

Smoking is linked to vascular problems and inflammation, which can negatively impact brain health.

#### Obesity

Obesity is associated with various cardiovascular risk factors that may contribute to cognitive decline.

#### Physical inactivity

Lack of regular physical activity can increase the risk of multiple health issues, including cognitive decline.

#### High cholesterol

Elevated cholesterol levels can lead to atherosclerosis and reduced blood flow to the brain, affecting cognitive function.

#### Family history

A family history of stroke or cognitive impairment may increase an individual’s susceptibility to these conditions.

#### Depression

Depression can negatively affect cognitive function, and individuals with depression may be at a higher risk of developing cognitive impairment.

#### Substance abuse

Chronic substance abuse can harm brain function and may accelerate cognitive decline.

#### Cardiovascular disease

Various heart-related conditions can affect blood flow to the brain and contribute to cognitive impairment.

#### Sleep disorders

Sleep disturbances like sleep apnea may affect cognitive decline.

Understanding these risk factors and their impact on cognitive health can help develop strategies to manage and mitigate the risk of cognitive decline after a stroke.

## Conclusion

Stroke and cognitive impairment are closely linked, with cognitive deficits being a common consequence of stroke. Understanding the connection between stroke and cognitive impairment is crucial for effectively managing post-stroke symptoms and optimizing patient outcomes. This abstract provided an overview of the background, methods, results, and conclusions related to stroke and cognitive impairment.

The background section highlighted the significant impact of stroke on cognitive function, emphasizing the prevalence and severity of cognitive impairments following stroke. The methodology section outlined the various investigations and diagnostic tools used to assess cognitive impairment in stroke patients, including neuroimaging, cognitive tests, and clinical assessments.

The results section summarized the key findings related to the epidemiology, pathophysiology, management, TOAST classification, prognosis, and comorbidities of stroke and cognitive impairment. It highlighted the high prevalence of cognitive impairment after stroke, the multifactorial nature of cognitive deficits, and the importance of managing comorbidities to optimize stroke outcomes. The TOAST classification provided valuable insights into the etiology and subtypes of stroke, aiding in individualized treatment strategies.

Furthermore, the conclusion underscores the importance of early diagnosis, comprehensive management, and targeted interventions for stroke and cognitive impairment. It emphasizes the need for a multidisciplinary approach involving healthcare professionals from various specialties to address the complex nature of cognitive deficits and comorbid conditions associated with stroke.

## Ethical approval

Ethics approval was not required for this review for the following reasons:

Ethics statement: It was exempted and waived at my institution.

Nature of the study: The review is a literature-based analysis not involving primary data collection from human subjects. Instead, it relies on the analysis and synthesis of existing published material.

Confidentiality and anonymity: As the review does not involve direct contact with human participants, there are no concerns regarding confidentiality, privacy, or the handling of personal data.

Minimal risk: The review poses minimal or no risk to human participants as it does not involve interventions, experiments, or direct interaction with individuals. The analysis focuses solely on previously published information.

Given these factors, the Institutional Review Board (IRB) of Mayo Clinic (IRB ID: 21-007698) has determined that ethics approval is not required for this review. The waiver was granted based on the ethical guidelines and policies outlined by the institution to ensure the protection of human subjects in research.

## Consent

Informed consent was not required for this article due to the use of publicly available information and data, which was obtained and analyzed in an aggregated and de-identified manner. The study did not involve any direct interaction or intervention with human subjects, and the research findings were based solely on existing public knowledge and data sources. Therefore, no personal information or individual participation was involved, eliminating the need for informed consent.

## Sources of funding

All authors have declared that they have no financial relationships at present or within the previous 3 years with any organizations that might have an interest in the submitted work.

## Author contribution

The author played important roles to actualize this article as shown below:

C.E.: conceptualization, visualization, supervision, oversight and leadership, and writing of the original draft.

## Conflicts of interest disclosure

In compliance with the ICMJE uniform disclosure form, all authors declare the following. Payment/services info: All authors have declared that no financial support was received from any organization for the submitted work. Other relationships: All authors have declared that there are no other relationships or activities that could appear to have influenced the submitted work.

## Research registration unique identifying number (UIN)

Not applicable.

## Guarantor

Chukwuka Elendu; E-mail: elenduchukwuka@yahoo.com.

## Data availability statement

Data will be made available by the authors upon reasonable request.

## Provenance and peer review

Commissioned and externally peer-reviewed.
